# Maintaining Epigenetic Inheritance During DNA Replication in Plants

**DOI:** 10.3389/fpls.2016.00038

**Published:** 2016-02-02

**Authors:** Francisco M. Iglesias, Pablo D. Cerdán

**Affiliations:** ^1^Fundación Instituto Leloir, IIBBA-CONICET Buenos Aires, Argentina; ^2^Facultad de Ciencias Exactas y Naturales, Universidad de Buenos Aires Buenos Aires, Argentina

**Keywords:** epigenetic inheritance, DNA replication, nucleosome assembly, DNA polymerases, plant development, *Arabidopsis thaliana*

## Abstract

Biotic and abiotic stresses alter the pattern of gene expression in plants. Depending on the frequency and duration of stress events, the effects on the transcriptional state of genes are “remembered” temporally or transmitted to daughter cells and, in some instances, even to offspring (transgenerational epigenetic inheritance). This “memory” effect, which can be found even in the absence of the original stress, has an epigenetic basis, through molecular mechanisms that take place at the chromatin and DNA level but do not imply changes in the DNA sequence. Many epigenetic mechanisms have been described and involve covalent modifications on the DNA and histones, such as DNA methylation, histone acetylation and methylation, and RNAi dependent silencing mechanisms. Some of these chromatin modifications need to be stable through cell division in order to be truly epigenetic. During DNA replication, histones are recycled during the formation of the new nucleosomes and this process is tightly regulated. Perturbations to the DNA replication process and/or the recycling of histones lead to epigenetic changes. In this mini-review, we discuss recent evidence aimed at linking DNA replication process to epigenetic inheritance in plants.

## Introduction

A myriad of studies have shown that plants are exposed to stress episodes, such as cold, heat and drought, that lead to transcriptional reprogramming of gene expression and phenotypic changes. This reprogramming can be “memorized” for short as well as for long periods of time, enabling plants to aid responses when these events recur. The former depends on the half-life of stress-induced proteins, RNAs and metabolites, whereas the latter depends on epigenetic processes. Epigenetic inheritance of changes in gene function means that they: (i) are mitotically and/or meiotically heritable; (ii) cannot be explained by changes in the DNA sequence; and (iii) are maintained without the influence of the initial *stimulus*. Memory occurs by multiple mechanisms, but requires chromatin changes, such as DNA methylation, histone modifications and non-coding RNAs. However, whether these chromatin modifications are the cause of heritable transcriptional changes remains controversial. In this mini-review, we discuss recent evidence relating the coupling of epigenetic inheritance with DNA replication in plants. On the other hand, how the replication of DNA is influenced by the chromatin landscape has been recently reviewed (Sequeira-Mendes and Gutierrez, 2015).

## Nucleosome Assembly at the Replication Fork Links DNA Replication with Epigenetic Inheritance

**FIGURE 1:**
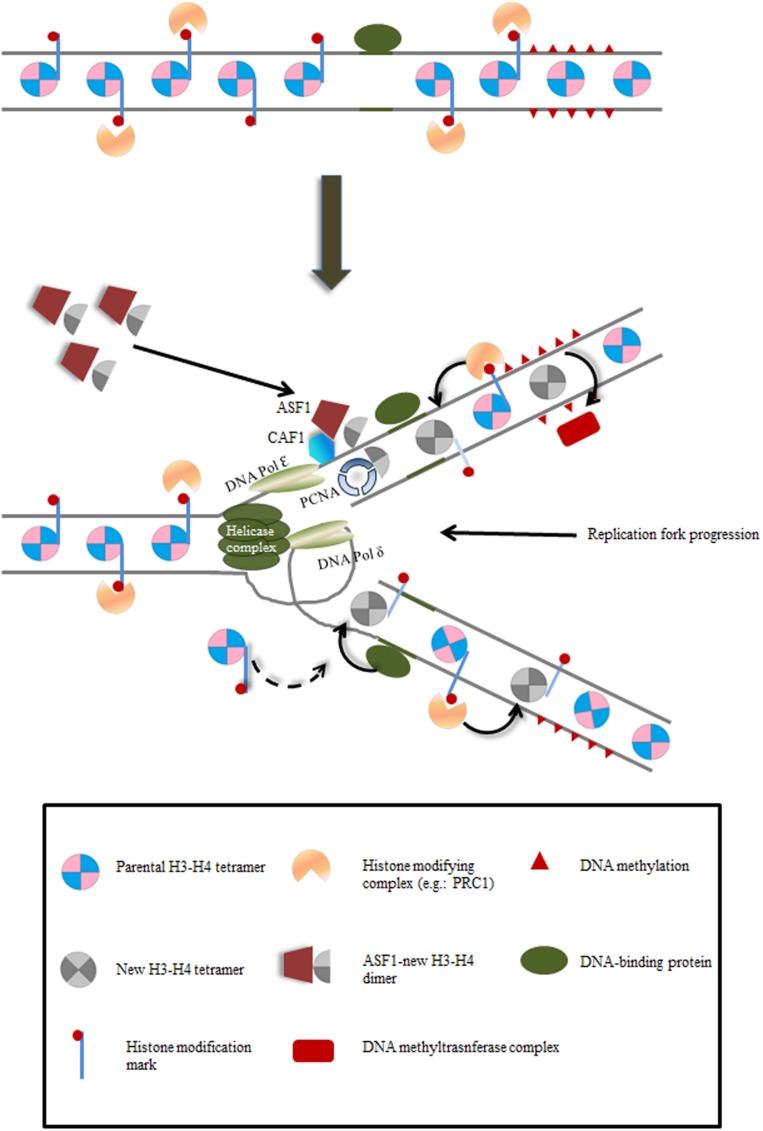
**Schematic depiction of mechanisms by which epigenetic inheritance may be maintained during DNA replication.** As replication fork proceeds, (i) newly deposited histones are modified by protein complexes (e.g.: PRC2) targeted by inherited modifications on parental histones; (ii) DNA binding proteins recruit/repel modification protein complexes; (iii) cross-talk between the two complementary mechanisms is also possible. For simplicity, H3–H4 nucleosomes are depicted.

The nucleosome is the basic chromatin unit, containing about 147 bp of DNA wrapped around an octameric protein core composed of two subunits of each histone: H2A, H2B, H3 and H4 (Luger et al., 1997). This octameric structure is disassembled in front of the advancing replication fork as two H2A–H2B dimers and one (H3–H4)_2_ tetramer and reassembly occurs behind the replication fork on both leading and lagging strands with both parental and newly synthesized histones (Gruss et al., 1993). The replication machinery has been reviewed elsewhere (Sequeira-Mendes and Gutierrez, 2015). The newly synthesized H3–H4 dimers are transported into the nucleus by the ANTI-SILENCING FUNCTION1 (ASF1) chaperone and then transferred to the CAF-1 chaperone. CAF-1 is bound to PROLIFERATING CELL NUCLEAR ANTIGEN (PCNA) at the replication fork, where the formation of the novo (H3–H4)_2_ tetramers takes place after successive transfer of H3–H4 dimers (**Figure 1**) (Wittmeyer and Formosa, 1997; Tyler et al., 1999; Wittmeyer et al., 1999; Moyer et al., 2006; Groth et al., 2007; Abe et al., 2011; Huang et al., 2013b; MacAlpine and Almouzni, 2013). The newly synthesized H4 is acetylated at K5 and K12 positions, which is a highly conserved histone modification, whereas the residues of H3 acetylation differ among species (Annunziato, 2013). Recent evidence sheds light on the segregation of parental nucleosomes at the replication fork. In yeast, the FACILITATES CHROMATIN TRANSCRIPTION complex (FACT) and the MINICHROMOSOME MAINTENANCE COMPLEX COMPONENT 2 (Mcm2) act together during their association with the replisome to bind and manage the parental histone complexes released from chromatin (Foltman et al., 2013). In this way, deposition is closely coupled to the replication machinery, as nucleosomes re-appear about 400 bp behind the replication fork on both leading and lagging strands (Radman-Livaja et al., 2011; Smith and Whitehouse, 2012). Hence, pre-existing parental nucleosomes contribute to approximately half of the histones on nascent DNA, suggesting an important contribution to the epigenome of daughter cells (Radman-Livaja et al., 2011; Alabert and Groth, 2012; Sarkies and Sale, 2012; Whitehouse and Smith, 2013; Campos et al., 2014). Chromatin modifications decorating the nucleosome core are thus believed to serve as carriers of epigenetic information.

Far from being complete, the understanding of this process in plants has been possible through diverse genetic screens in *Arabidopsis* (Liu and Gong, 2011). CAF-1 is a highly conserved complex in eukaryotes. It is recruited by PCNA to the replication fork and is involved in supplying *de novo* synthesized H3–H4 dimers to the DNA replication machinery (**Figure 1**). *Arabidopsis* CAF-1 is composed of three subunits encoded by *FASCIATA1 (FAS1)*, *FASCIATA2 (FAS2)* and *MULTICOPY SUPPRESSOR OF IRA1 (MSI1)* (Kaya et al., 2001; Kirik et al., 2006; Ono et al., 2006; Ramirez-Parra and Gutierrez, 2007). Mutants in *FAS1, FAS2* and *MSI1* show severe developmental defects, such as smaller leaves, fasciated stems, abnormal phyllotaxis, reduced fertility, more open euchromatin and reduced heterochromatin content. The *Arabidopsis ASF1* homologues *AtASF1A* and *AtASF1B* have an important role during development. In contrast to single mutants, the double mutant *asf1a asf1b* shows severe defects in growth and vegetative and reproductive development, together with constitutive activation of DNA replication stress genes (Zhu et al., 2011). In addition, they have a role in the heat shock response, by participating in the activation of heat stress genes, supposedly through nucleosome removal and H3K56ac accumulation (Weng et al., 2014). The role of the primary chaperone of H2A–H2B dimers, NUCLEOSOME ASSEMBLY PROTEIN1 (NAP1), is conserved in Eukaryotes (Burgess and Zhang, 2013). In *Arabidopsis*, NAP1-RELATED PROTEIN 1 and 2 (NRP1 and NPR2) chaperone *de novo* synthesized H2A and H2B to be assembled into the nucleosomes during replication of DNA and they are also required for the formation of heterochromatin (Zhu et al., 2006).

Topoisomerases break one or both DNA strands to relax DNA conformation, and mutant analysis suggested they have a role in the maintenance of chromatin structure. Loss of function mutants of topoisomerase MGOUN1 (TOP1-β) displayed a stochastic occurrence of ectopic *AGAMOUS* (*AG*):*GUS* expression, suggesting that maintenance of chromatin marks is unstable in the absence of topoisomerase function, allowing random switches between on/off states. Once established, such expression states appear to be copied to daughter cells during cell divisions, but occasionally switch back to a repressed state as evidenced by revertant *AG:GUS* sectors (Graf et al., 2010). Dimethylation of Lysine 9 of H3 (H3K9me2) and DNA methylation are reduced in plants treated with camptothecin, an inhibitor of the DNA topoisomerase 1α (TOP1α), and in *top1a* loss of function mutants, leading to the de-repression of Pol V target loci (Dinh et al., 2014). In agreement to that, in studies performed with the *top1α-2* allele, it was demonstrated that TOP1α is required for the deposition of H3 trimethylated on K27 (H3K27me3) at PcG target genes (Liu et al., 2014). However, a simple relationship between TOP1α and gene repression was not found since another subgroup of PcG genes was downregulated in *top1α-2* mutants. It was also shown that TOP1α is important to decrease nucleosome density and hence, TOP1α could act as a promoter of gene expression in a subset of genes (Liu et al., 2014).

## DNA Replication Dynamics and Epigenetics

In *Arabidopsis*, as in other eukaryotes, three replicative DNA polymerases duplicate DNA: Polα (with primase activity), Polε (leading strand extension) and Polδ (lagging strand extension). Their catalytic subunits are encoded by *POLA1* (also known as *ICU2*), *POLE1* and *POLD1* and their roles in epigenetic inheritance were evidenced in different genetic screenings. The *polα*/*icu2-1* missense mutation leads to reactivation of a silenced *35S-NPTII* transgene and repetitive elements due to decreased H3K9me2, but not DNA methylation (Liu et al., 2010). *icu2-1* interacts genetically with *CURLY LEAF* (*CLF*), which encodes a SET-domain component of Polycomb Repressive Complex 2 (PRC2) which methylates H3K27, and also interacts with *FAS1* and with *LIKE-HETEROCHROMATIN PROTEIN 1* (*LHP1*), which encodes a protein with PRC1 activity in *Arabidopsis* (Barrero et al., 2007). POLA/ICU2 is required for the stable inheritance of vernalization memory through mitoses. The flowering repressor *FLOWERING LOCUS C (FLC)* is stably repressed after vernalization. However, the *icu2-1* allele induces a mosaic de-repression of an *FLC:GUS* marker after vernalization (Hyun et al., 2013). A similar mosaic de-repression was also shown for an *AG:GUS* marker (Barrero et al., 2007). The variability in the degree of derepression was explained by the role of POLA/ICU2 in the mitotic inheritance of the H3K27me3 marks and further, the *icu2-1* allele led to reduced binding of LHP1 at target loci (Hyun et al., 2013). The similarities among mutants affected in chromatin assembly factors and topoisomerases with mutants in POLα/ICU2 underscores its role in the process of chromatin assembly and the maintenance of repressive histone marks through mitosis.

Mutants for the POLE1 and POLD1 catalytic subunits have also been described. *early in short days7-1 (esd7-1)* (del Olmo et al., 2010) and *ABA overly sensitive 4-1* (*abo4-1*) (Yin et al., 2009) are hypomorphic alleles of *POLE*. *gigantea suppressor5 (gis5)* (Iglesias et al., 2015) and the *polδ1* (Schuermann et al., 2009) are hypomorphic or RNA interference alleles of *POLD*. They share similar developmental abnormalities, display early flowering and leaf incurvature. *POLE/ESD7* interacts genetically with *PRC2* and *PRC1* genes, shows more severe phenotypes when combined with *clf* or *tfl2* mutants (del Olmo et al., 2010). In addition, POLE/ESD7 interacts with TFL2 *in vitro*. These findings agree with the fact that discrete regions at the *FLOWERING LOCUS T* (*FT)* chromatin are enriched in H3K4me3 and H3 acetyation (H3Ac) in *esd7* and *abo4-1* mutants, and the *FLC* first intron is enriched in H3K27me3 in *abo4-1* mutants, correlating with higher *FT* and lower *FLC* expression levels (del Olmo et al., 2010).

In *gis5*, early flowering and curly leaves correlate with higher levels of H3K4me3 and H3Ac, and higher expression of *SEP3* in phloem cells. Furthermore, as the *gis5* allele is thermosensitive, the strong increase in *SEP3* expression and H3K4me3 at the *SEP3* locus were both temperature-dependent and correlated well with the DNA replication stress responses and the increase in homologous recombination (HR). Taking into account that the *gis5* mutation affected an Ala residue poorly accessible to the solvent and close to the active site, the changes in *SEP3* epigenetic marks and expression could be produced by the changes in the dynamics of DNA replication, a hypothesis that needs further testing (Iglesias et al., 2015).

## Epigenetic Inheritance Relies on Histone and Non-Histone Proteins

The evidence supporting the link between changes in chromatin marks and epigenetic inheritance is mainly based on correlations between changes in gene expression patterns and changes in the abundance of chromatin modifications. Nevertheless, correlation does not mean cause and therefore, the changes in chromatin states may reflect the transcriptional level rather than be the cause of it (Henikoff and Shilatifard, 2011). If chromatin marks are truly epigenetic, looking at parental histones distribution during the replication of DNA should give an insight into the mechanisms of epigenetic inheritance (**Figure 1**).

As H3–H4 tetramers are twofold diluted and are inherited randomly at each daughter strand, chromatin marks on this nucleosomes are considered to contribute to the inheritance of chromatin states and somehow inherited during DNA replication. This contribution may be related to: (i) the extension of the domain (number of nucleosomes enriched with the chromatin marks), (ii) the turnover of histones modifications during the cell cycle, (iii) the recognition and binding of chromatin marks on the parental (H3–H4)_2_ tetramers by the protein complexes involved in their own deposition, (iv) physical interaction with the DNA replication machinery, and (v) the kinetics for the reestablishment of chromatin marks on newly deposited histones (Sarkies and Sale, 2012). In this regard, H3K27me3, H3K9me3 and DNA methylation have been proposed to contribute themselves to the maintenance of epigenetic inheritance (Huang et al., 2013a).

Interactions between the DNA replication machinery and protein complexes involved in the deposition of chromatin modifications are necessary for the maintenance of the chromatin states (Margueron and Reinberg, 2010; Alabert and Groth, 2012). During DNA replication, nascent DNA is hemimethylated. In animals, parental DNA methylation is reestablished at CG motifs in daughter strands by recruiting the maintenance DNA-methylase DNMT1 to the replication fork by PCNA and UHRF1 (Bostick et al., 2007; Woo et al., 2008). Also, CAF1 and UHRF1 recruit H3K9 methyltransferase at the PCNA to monomethylate H3K9 at heterochromatin domains (Esteve et al., 2006; Scharf et al., 2009; Xu et al., 2012).

In plants, VIM proteins (the orthologs of mammalian UHRF proteins) recruit MET1, the ortholog of mammalian DNMT1, suggesting that plants share a similar mechanism with mammals for CG reestablishment (Woo et al., 2008). In *Arabidopsis*, the CHROMOMETHYLASE 3 (CMT3) is involved in the maintenance of CHG methylation and is recruited by H3K9me2 (Bartee et al., 2001; Lindroth et al., 2004). On the other hand, the histone methyltransferase KYP/SUVH4 binds, and is recruited onto, methylated cytosines (Johnson et al., 2007; Du et al., 2012; Johnson et al., 2014), showing the existence of a positive feedback loop between DNA methylation and histone marks in plants (**Figure 1**).

Recently, a direct physical and functional link between DNA replication, small RNA generation and H3K9 dimethylation was shown to occur in yeast. The Cdc20 subunit of Polε is conserved among eukaryotes. Cdc20 interacts directly with the silencing factor Dos2 forming a complex with Mms19, which is a transcriptional activator that promotes heterochromatin transcription by Pol II, a necessary step to establish heterochromatin silencing by H3K9 methylation during DNA replication (Li et al., 2011). If a similar process exists in plants is currently unknown, but a Polε mediated mechanism of recruiting silencing factors is possibly present in *Arabidopsis*. It has been found that LHP1 interacts with ICU2/POLA and ESD7/POLE subunits (Barrero et al., 2007; Yin et al., 2009; Hyun et al., 2013), as discussed above. Contrary to other systems, plants possess two other RNA Polymerases, RNA PolIV and RNA PolV, which are involved in DNA silencing. Recently, a link between HISTONE DEACETYLASE6 (HDA6) and MET1 has been established and helps to describe a truly epigenetic process (Blevins et al., 2014). In *hda6* mutants a subset of loci are derepressed at similar levels than in *polIV* single and *hda6 polIV* double mutants suggesting that both are in the same pathway for the silencing of these groups of genes (Blevins et al., 2014). However, *POLIV* transgenes complement the *polIV* mutants whereas *HDA6* transgenes do not complement *hda6* mutants only for this specific subset of genes, suggesting the loss of epigenetic information at these loci, which cannot be restored after complementation with *HDA6*. This epigenetic information is maintained by MET1 and HDA6 by conferring a silent status to these *loci* which depend on certain level of GC methylation. Further downstream, the silent status is somehow recognized to ensure Pol IV recruitment and RNA dependent DNA methylation to further silencing (Blevins et al., 2014). It is speculated that HDA6 is necessary for erasing acetyl groups on newly deposited histones, before recruiting Pol IV and MET1 to methylate DNA.

These examples show that a close interrelationship between chromatin modifications, chromatin modifiers and the DNA replication machinery is required for proper perpetuation of the chromatin states. Moreover, this association is dynamic. Recent comprehensive studies from mammals and *Drosophila* systems, using nascent chromatin capture (NCC) to profile chromatin proteome dynamics from newly synthesized DNA, show association dynamics for about 4,000 proteins when comparing nascent chromatin with mature post-replicative chromatin (Alabert et al., 2014). The authors suggested that histone modifications, such as H3K9me3 and H3K27me3 serve as truly epigenetic marks, given their levels are relatively stable from duplicating to maturing chromatin. On the other hand, it has been shown that there is no evidence for these histone modifications to be considered themselves truly epigenetic marks given their low levels in the nascent chromatin (about 400 bp from the replication fork) (Petruk et al., 2013). These results agree with previous reports in *Drosophila* showing that H3K27me3 levels are reestablished at G1/M phase (Lanzuolo et al., 2011). Nevertheless, there is consensus from both studies to consider trithorax (TrxG) and PcG complexes as truly epigenetic carriers, as they are in close proximity to the replication fork (Follmer et al., 2012; Fonseca et al., 2012; Lo et al., 2012; Petruk et al., 2012). Similar approaches are not currently available for plant systems.

The inheritance of the vernalized state is a model of epigenetic memory in plants. In plants exposed to long periods of cold, *FLC* chromatin is enriched in H3K27me3 and PRC1 complex, which stably silence the *FLC* chromatin (Xiao and Wagner, 2015). This process requires the PRC2 complex to spread H3K27me3 across the whole *FLC* locus. This takes place when H3K27me3 is recognized by PRC2. During DNA replication, parental nucleosomes with this histone modification recruit PRC2 proteins which catalyze the trimethylation on neighboring, H3K27me3 depleted nucleosomes (Hansen et al., 2008; Margueron et al., 2009). Similar spreading from the initial nucleation site to adjacent sequences has been observed for reporter systems in plants (see above). Thus, at both *AG:GUS* and *FLC:GUS* transgenic lines strong enrichment of H3K27me3 was found not only at both *AG* (and *FLC*) but also at GUS sequences (Hyun et al., 2013). It was proposed that a buffer system helps to overcome the fluctuations in the levels of epigenetic marks on target loci, especially when these marks are diluted during DNA replication, and then restored during the maturation of chromatin (Huang et al., 2013a). On the other hand, it is unlikely that every single histone modification will be self-copied. There is evidence that supports the existence of primary histone modifications that influence the inheritance of “secondary” histone modifications, as suggested for monomethylated forms of H3K4, H3K9 and H3K27 with respect to the trimethylated forms (Sarkies and Sale, 2012).

PRC2 and TRXG protein complexes bind DNA sequences known as Polycomb (PRE) and Trithorax (TRE) response elements, respectively (Margueron and Reinberg, 2010). By binding DNA sequence targets they contribute to inhibit (PRC2) or activate (TRXG) gene transcription (Steffen and Ringrose, 2014). Recent mechanistic evidence in *Arabidopsis* suggests that cell division provides a window of opportunity to change fate. The flower development protein AG induces *KNUCKLES* (*KNU*) expression by displacing PcG proteins from PRE sequences on *KNU* locus, a process that takes about 2 days to be completed, leading to a failure in the maintenance of repressive histone modifications (Sun et al., 2014). It has been recently demonstrated in yeast that strongly bound transcription factors that remain associated to DNA cognate sites during DNA replication can influence nucleosome organization during the maturation of Okazaki fragments by Poldδ (Smith and Whitehouse, 2012). Interestingly, the VERNALIZATION1 transcription factor, which is involved in epigenetic regulation of *FLC*, remains associated with euchromatin during mitosis (King et al., 2013).

In summary, plants are an interesting model system to study the impact that DNA replication dynamics has on the maintenance of epigenetic inheritance due to the high conservation of the DNA replication machinery among eukaryotes and the high tolerance of plants to missense mutations on DNA replication components. Additional studies tending to investigate the functional and biochemical relationship between DNA polymerases, DNA replication factors and protein complexes that modify chromatin will provide further insights into DNA replication-coupled epigenetic inheritance.

## Author Contributions

All authors listed, have made substantial, direct and intellectual contribution to the work, and approved it for publication.

## Conflict of Interest Statement

The authors declare that the research was conducted in the absence of any commercial or financial relationships that could be construed as a potential conflict of interest.
